# Comprehensive analysis of lncRNA–miRNA–mRNA during proliferative phase of rat liver regeneration

**DOI:** 10.1002/jcp.28529

**Published:** 2019-03-27

**Authors:** Haijing Bai, Jianlin Guo, Cuifang Chang, Xueqiang Guo, Cunshuan Xu, Wei Jin

**Affiliations:** ^1^ College of Life Science, Henan Normal University Xinxiang Henan China; ^2^ State Key Laboratory Cultivation Base for Cell Differentiation Regulation Xinxiang Henan China

**Keywords:** high‐throughput sequencing technology, lncRNA, miRNA, mRNA, rat liver regeneration

## Abstract

This study aims to reveal the regulatory mechanism of lncRNAs–miRNAs–mRNAs network during the proliferative phase of liver regeneration (LR). High‐throughput sequencing technology was performed, and a total of 1,738 differentially expressed lncRNAs (DE lncRNAs), 167 known differentially expressed miRNAs (DE miRNAs), and 2,727 differentially expressed mRNAs were identified. Then, the target DE lncRNAs and DE mRNAs regulated by the same miRNAs were screened and a ceRNA regulatory network containing 32 miRNAs, 107 lncRNAs, and 270 mRNAs was constructed. Insulin signaling pathway, pyrimidine metabolism, axon guidance, carbohydrate digestion and absorption, and pyruvate metabolism were significantly enriched in the network. Through literature review and the regulatory relationship between lncRNAs and miRNAs, nine core lncRNAs were identified, which might play important roles during the proliferative phase of rat LR. This study analyzed lncRNA–miRNA–mRNA regulatory network for the first time during the proliferative phase of rat LR, providing clues for exploring the mechanism of LR and the treatment of liver diseases.

## 1 INTRODUCTION

The liver is one of the important organs in human and animal, which is responsible for a variety of physiological functions. It has a strong ability of regeneration after liver loss or toxic injury. Liver regeneration is a highly organized tissue growth process of restoring the original framework structure and tissue specific function after liver injury (Jeon et al., 2013). It was usually divided into three phases including initiation, proliferation, and termination. In the proliferative phase, the main process is the proliferation of hepatocyte, which replicate once or twice under the synergistic action of various growth factors and inflammatory cytokines. Long noncoding RNA (lncRNA) is a class of RNA molecules with a length of more than 200 nucleotides (nt) and lacking an open reading frame that plays crucial roles in epigenetics, transcriptional regulation, and posttranscriptional regulation (Maruyama & Suzuki, 2012). Studies have shown that lncRNAs were not only involved in normal physiological activities but also related to the occurrence and development of various tumors (Chen et al., 2013). MicroRNA (miRNA) is a class of endogenous noncoding single‐stranded RNA molecules with a length of approximately 22 nt. It plays important roles in regulating the expression of messenger RNAs (mRNAs) through specifically binding to the 3′‐untranslated region (3′‐UTR) of the encoding gene. There is evidence that miRNAs can regulate a variety of cell processes and developmental processes (Krol, Loedige, & Filipowicz, 2010). Salmena, Poliseno, Tay, Kats & Pandolfi (2011) proposed a competition endogenous RNA (ceRNA) hypothesis, which pointed that mRNA, transcriptional pseudogenes and long noncoding RNA could communicate to each other through their ability to compete for microRNA binding using microRNA response elements (MREs) (Wang, Zhang, He, & Gou, 2018; Salmena et al., 2011). Subsequently, increasing evidence indicated that lncRNAs, as ceRNA, were associated with a variety of cancers, including hepatocellular carcinoma (HCC)( Wang et al., 2015). Accordingly, it is necessary to explore the regulatory network of lncRNA–miRNA–mRNA during the proliferative phase of liver regeneration (LR). In present study, high‐throughput sequencing technology was performed to obtain the miRNA, mRNA, and lncRNAs expression data during the proliferative phase of rat LR and lncRNAs–miRNAs–mRNAs regulatory network was established. Our findings might lay the foundation for further investigate the lncRNAs–miRNAs–mRNAs interaction network during LR and liver‐associated diseases.

## 2 MATERIALS AND METHODS

### 2.1 Preparation of rat LR model after 2/3 hepatectomy

The healthy adult male Sprague–Dawley (SD) rats weighing 210–250 g were provided by Laboratory Animal Center of Zhengzhou University (Zhengzhou, China). These rats were raised in a controlled temperature room 19–23℃ with a relative humidity 50–70% and illumination time 12 hr/day (8:00–20:00), and permitted to freely have water and food. A total of 36 rats were randomly divided into six groups with six rats per group: Five partial hepatectomy (PH) groups and one normal group (CG). The rats in PH group were subjected to 2/3 PH in accordance with the method of Xu C. et al. (2010). They were anesthetized and killed at 0, 12, 24, 30, 36, and 72 hr after surgery. The right liver lobe was mixed each time point of six rats and restored in −80℃. All operations conformed to the Animal Protection Law of China and Animal Ethics.

### 2.2 Sequencing of lncRNA and mRNA and identification of DE lncRNA and DE mRNA

The mirVana miRNA Isolation Kit (Ambion) was used to extract total RNA and the TruSeq Stranded Total RNA with Ribo‐Zero Gold was used to construct the complementary DNA (cDNA) libraries. In brief, after total RNA extracted and ribosomal RNA digested, the RNA was broken into short fragments by the interrupt reagent. The first cDNA chain was synthesized using these short fragments as template and a random six‐base as primer. Then the second cDNA chain was synthesized using the first cDNA as template and the dTTP was replaced with dUTP. After repairing the end, jointing adenylate 3′ ends and sequence adapters, the second cDNA chain was digested by UNG (Uracil‐N‐Glycosylase) enzyme, and the first cDNA with different joints was retained. Agarose gel electrophoresis was used to select the fragment size. Finally, polymerase chain reaction (PCR) amplification was performed. After the constructed library passed the quality inspection with Agilent 2100 Bioanalyzer (Agilent Technologies, Santa Clara), Illumina sequencing platform (Hiseq X Ten) was used for sequencing.

Raw reads of fastq format were performed quality preprocessing by using Trimmomatic (0.36). After wiping off the adapter and low‐quality reads, the clean reads were obtained. The hisat2 (2.2.1.0) was use to align the clean reads with the rat reference genome. For unmapped reads, transcripts were reconstructed based on the probability model based on the comparison results of each sample by using StringTie (1.3.3b). Finally, the candidate lncRNAs were identified with software CPC (0.9‐r2), CNCI (1.0), PFAM (v30), and PLEK (1.2), which were used to predict the coding capacity of the transcripts. The expression abundance of lncRNAs and mRNAs were measured by FPKM. The differentially expressed mRNAs (DE mRNAs) and differentially expressed lncRNAs (DE lncRNAs) were detected by the negative binomial distribution test based on the DESeq package (1.18.0). The fold change ≥ 2 or fold change ≤ 0.5, and *p* value < 0.05 were used as the cut‐off criteria.

### 2.3 Sequencing of miRNA and identification of DE miRNA

The mirVana miRNA Isolation Kit (Ambion) and TruSeq Small RNA Sample Prep Kits were used to extract total RNA and construct the cDNA libraries. The whole process was carried out in strict accordance with the reagent instructions. First, T4 ligase was used to ligate a 5′ adapter and a 3′ adapter to the RNA molecules. Then a SuperScript II Reverse Transcription Kit (Invitrogen) was used to reverse‐transcribed 5′ and 3′ adapter‐ligated RNA to cDNA and PCR amplification was performed. Finally, the cDNA product was purified by RNA Gel Electrophoresis and gel recovery. The size and purity of the sample were determined using an Agilent 2100 Bioanalyzer (Agilent Technologies, Santa Clara). The Illumina sequencing platform was used for sequencing analysis.

Raw reads of fastq format were processed consisting of removing adapter and low‐quality reads including sequences with quality score less than 20 and sequences with N base to obtain high‐quality clean reads. The Bowtie2 was use to map the clean reads to mature miRNAs in miRBase 21.0 database. These consistent sequences were considered as the known miRNAs. The expression level of miRNAs was measured by TPM. The *p* value was calculated by Audic–Claverie statistic. The fold change ≥ 2 or fold change ≤ 0.5, and *p* value < 0.05 were used as the cut‐off criteria.

### 2.4 Function enrichment analysis

To analysis the biological function of lncRNAs, Gene Ontology (GO) enrichment analysis and Kyoto Encyclopedia of Genes and Genomes (KEGG) pathway analyses were performed on the DE mRNAs and predicted target genes of DE miRNA and DE lncRNAs using GeneCodis3 bioinformatics resources (http://genecodis.cnb.csic.es) and DAVID Bioinformatics Resources 6.8 ((https://david.ncifcrf.gov/). GO enrichment analysis included biological process (BP), molecular function (MF), and cellular component (CC). The *p* values have been obtained through hypergeometric analysis corrected by the false discovery rate (FDR) method. Both GO terms and KEGG pathways were considered to be significantly enriched with FDR < 0.05.

### 2.5 Construction of the ceRNAs regulatory network

Candidate mRNAs, an lncRNAs regulated by the DE miRNAs, were predicted by Shanghai OE biotech Co., Ltd using miRanda v3.3a. The Miranda algorithm was based on dynamic programming to comprehensively evaluate the target genes through miRNA–3′‐UTR sequencing matching and energy stability. The threshold parameter was set as described previously: S ≥ 150, ΔG ≤ −30 kcal/mol and strict 5′ seed pairing (Zhao et al., 2018). S refers to single residue pair match scores in the matching area, and ΔG refers to free energy when the double chains combine. Among these predicted DE miRNA–DE mRNA pairs, DE mRNAs opposite with their corresponding DE miRNAs in this study were defined as the targets of DE miRNA with high accuracy. Based on the DE miRNA–DE mRNA interaction analysis and DE miRNA–DE lncRNA interaction analysis, an lncRNA–miRNA–mRNA regulatory network was constructed and visualized by Cytoscape (v3.6.1) software.

### 2.6 Construction of TF–miRNA–lncRNA regulatory network

The mRNAs in the lncRNA–miRNA–mRNA interaction network was analyzed by AnimalTFDB database (http://bioinfo.life.hust.edu.cn/AnimalTFDB/#!/) to identify transcription factors (TF). Then a TF–miRNA–lncRNA regulatory network was constructed based on TF–miRNA and miRNA–lncRNAs interaction pairs and visualized by Cytoscape (v3.6.1) software.

## 3 RESULTS

### 3.1 DE mRNAs, DE miRNA, and DE lncRNAs during the proliferative phase of rat LR

**Figure 1:**
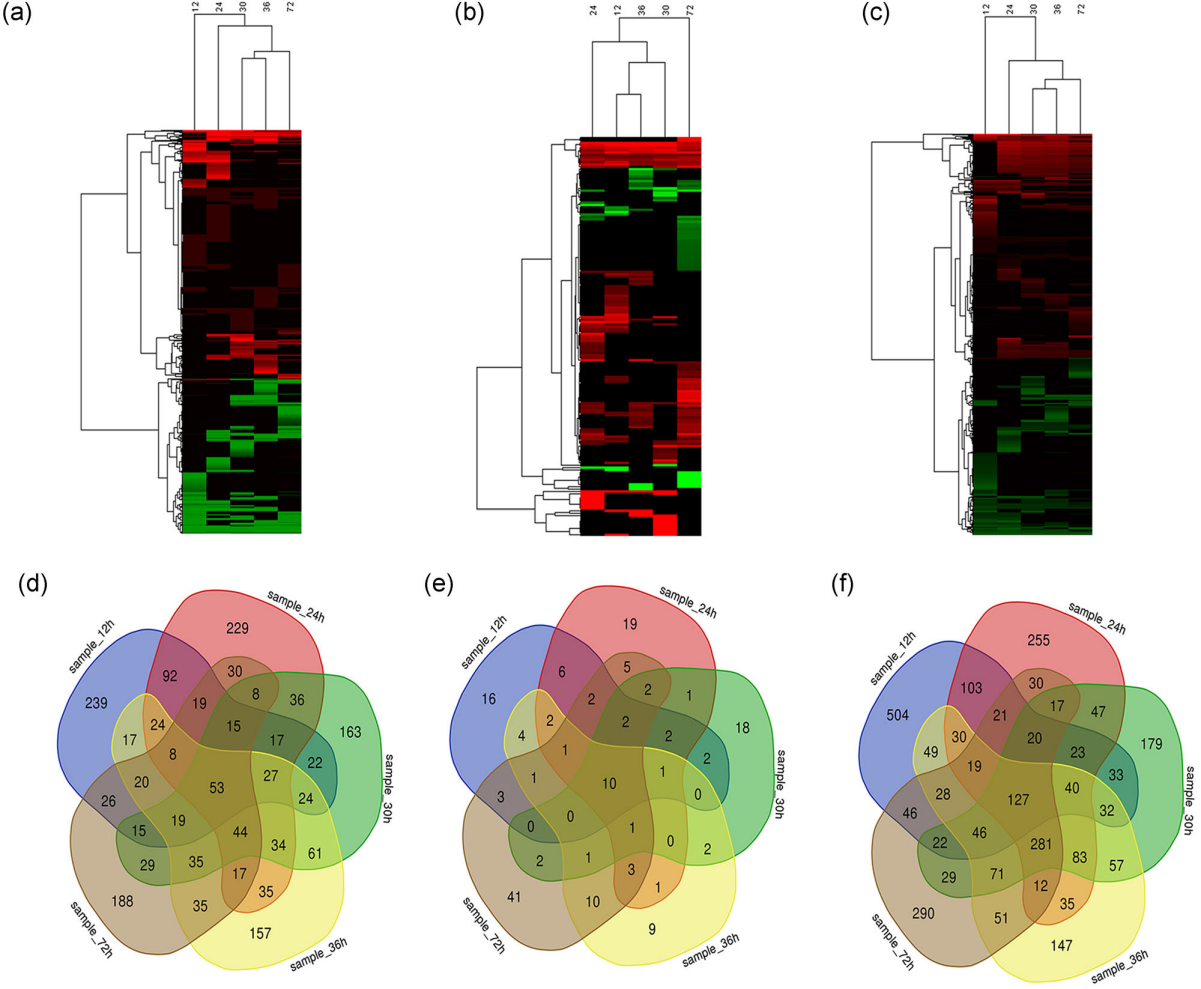
Expression pattern of lncRNAs, miRNAs, and mRNAs during the proliferative phase of rat LR. (a–c) Heatmap of DE lncRNAs, DE miRNAs, and DE mRNAs during the proliferative phase of rat LR. (d–f) Venn analysis of DE lncRNAs, DE miRNAs, and DE mRNAs detected at each time points. DE: differently expressed; lncRNAs: long noncoding RNAs; miRNAs: microRNAs; mRNAs: messenger RNAs [Color figure can be viewed at wileyonlinelibrary.com]

The expression profiles of lncRNAs, miRNAs, and mRNAs during the proliferative phase of rat LR were detected by high‐throughput sequencing technique. Compared with normal group (CG, 0 hr), a total of 1,738 DE lncRNAs were identified, with 1,069 upregulated, 653 downregulated, and 16 up/downregulated (*p* < 0.05) at 12, 24, 30, 36, and 72 hr after PH (Figure 1a; Additional file 1). Among them, 637 lncRNAs were detected at 12 hr after PH, 688 at 24 hr, 602 at 30 hr, 610 at 36 hr, and 561 at 72 hr (Figure 1d). A total of 167 known DE miRNAs was identified, with 113 upregulated, and 54 downregulated at 12, 24, 30, 36, and 72 hr after PH (*p* < 0.05; Figure 1b; Additional file 2). Among them, 52 miRNAs were detected at 12 hr after PH, 58 at 24 hr, 44 at 30 hr, 46 at 36 hr, and 84 at 72 hr (Figure 1e). A total of 2,727 DE mRNAs were identified, with 1,515 upregulated, 1,186 downregulated and 26 up/downregulated at 12, 24, 30, 36, and 72 hr after PH (*p* < 0.05; Figure 1c; Additional file 3). Among them, 1,143 mRNAs were detected at 12 hr after PH, 1,143 at 24 hr, 1,107 at 30 hr, 1,108 at 36 hr, and 1,110 at 72 hr (Figure 1f).

### 3.2 Function analysis of DE miRNAs, DE lncRNAs, and DE mRNAs during rat LR

Of the 167 DE miRNA, 20 miRNAs have been studied to be correlation with LR, such as miR‐127 (Pan et al., 2012), miR‐21 (Castro et al., 2010), miR‐34a (X. P. Wang et al., 2017), miR‐429 (C. Zhang et al., 2018), miR‐376b (Lu et al., 2015), miR‐382 (Bei et al., 2016), miR‐378 (Song et al., 2010), miR‐155 (Lin et al., 2018), miR‐25 (X. Xu et al., 2016), miR‐106b (X. Xu et al., 2016), miR‐133b (Gjymishka et al., 2016), miR‐125b (Hyun, Wang, Kim, Kim, & Jung, 2015), miR‐144 (Chaveles et al., 2012), miR‐451 (Chaveles et al., 2012), miR‐582‐3p (Chaveles et al., 2012), miR‐181c (Geng et al., 2016), miR‐183 (Geng et al., 2016), miR‐429 (Geng et al., 2016), miR‐27a (Ji et al., 2009), and miR‐30e (Ling et al., 2018). Unlike the DE miRNAs, most of the 1,738 DE lncRNAs were with unknown function. Functional analysis of DE mRNAs indicated that the most significant enriched BP were cell division, chromosome segregation, mitotic nuclear division, DNA replication, and oxidation‐reduction process, and so forth. Pathways in cell cycle, metabolic pathways, extracellular matrix (ECM)‐receptor interaction, focal adhesion, p53 signaling pathway, PI3K‐Akt signaling pathway, FoxO signaling pathway, and insulin signaling pathway may be closely enriched during the proliferative phase of rat LR.

### 3.3 Construction of lncRNA–miRNA–mRNA regulatory network during the proliferative phase of rat LR

The DE mRNAs targeted by DE miRNAs were predicted using miRanda algorithm according to the miRNA–mRNA binding data. Totally, 373 miRNA–mRNA target pairs removing duplicates were obtained which were consisted of 54 miRNAs and 307 mRNAs (Additional file 4). Five significant miRNAs, rno‐miR‐370‐3p (degree = 53), rno‐miR‐324‐3p (degree = 35), rno‐miR‐6315 (degree = 27), rno‐miR‐1956‐5p (degree = 25), and rno‐miR‐484 (degree = 25) had the most target mRNAs.

In the next step, the DE lncRNAs regulated by DE miRNAs were analyzed by miRanda algorithm. Totally, 116 miRNA–lncRNA regulatory pairs were identified including 34 miRNAs and 108 lncRNAs (Additional file 5). In the miRNA–lncRNA network, rno‐miR‐296‐3p (degree = 12), rno‐miR‐324‐3p (degree = 11), rno‐miR‐370‐3p (degree = 11), rno‐miR‐331‐3p (degree = 8), and rno‐miR‐6315 (degree = 8) had the most target lncRNAs.

**Figure 2:**
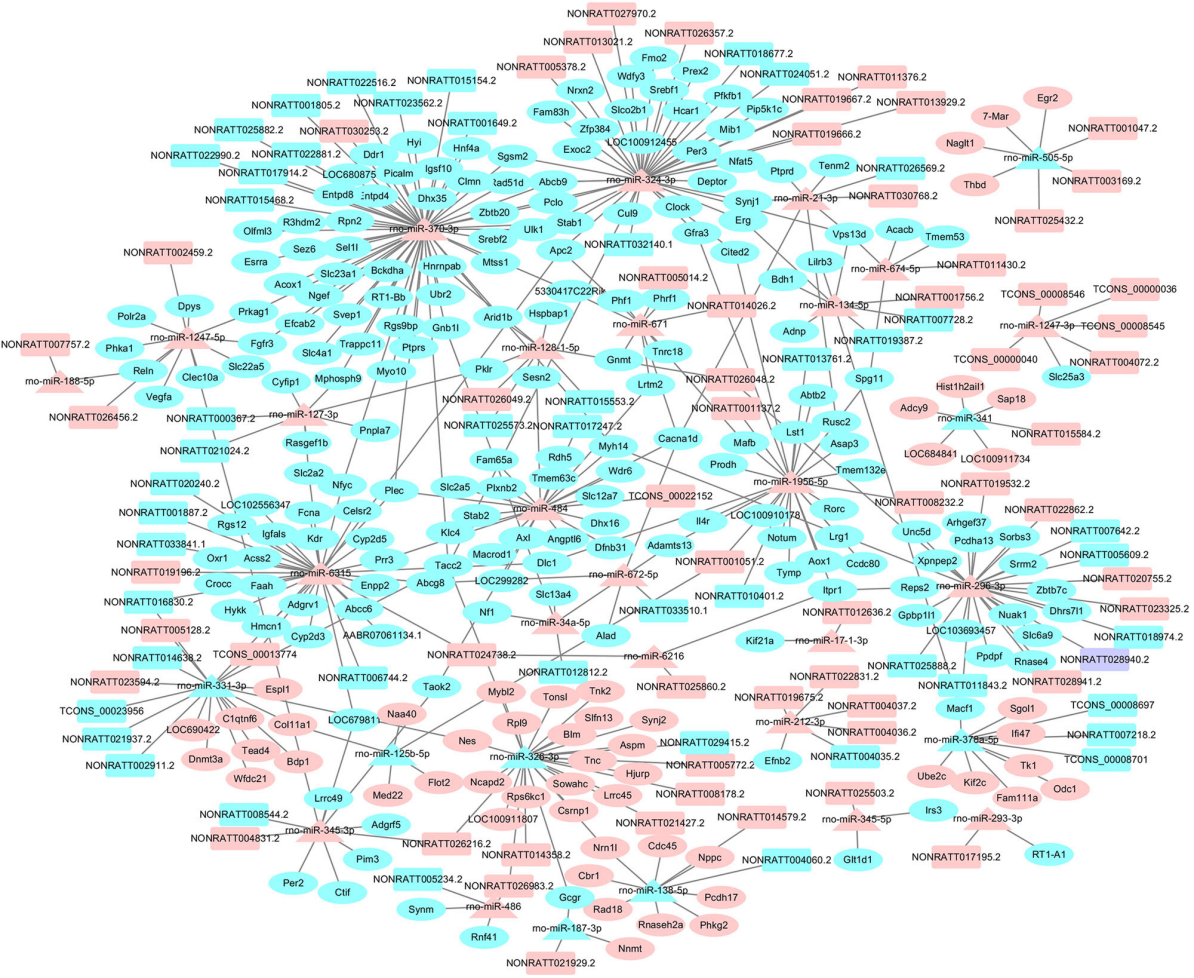
DE lncRNA–DE miRNA–DE mRNA interaction networks during the proliferative phase of rat LR. Rectangles, triangle, and ellipses represented DE lncRNAs, DE miRNAs, and DE mRNAs, respectively. Pink, light blue, and purple color represented upregulation, downregulation, and up/downregulation, respectively. DE: differently expressed; lncRNAs: long noncoding RNAs; miRNAs: microRNAs; mRNAs: messenger RNAs [Color figure can be viewed at wileyonlinelibrary.com]

Based on the regulatory pairs of miRNA–mRNA and miRNA–lncRNA, a lncRNA–miRNA–mRNA network was constructed, consisted of 32 miRNAs, 107 lncRNAs, and 270 mRNAs (Figure 2). Of these mRNAs, 63 were upregulated, and 207 were downregulated. Each mRNA or lncRNA could be regulated by one or more miRNA and vice versa.

### 3.4 Function annotation of candidate genes during proliferative phase of rat LR

First, gene ontology (GO) enrichment analysis of the 270 mRNAs was conducted to reveal their functions. The result showed that 86 BP terms, 19 MF terms, and 34 CC terms were enriched (Additional file 6). The most enriched BP terms were multicellular organismal development, anatomical structure development, cellular component organization, nitrogen compound metabolic process, and cellular metabolic process. As for MF, the most enriched GO terms were protein binding, nucleotide binding, sequence‐specific DNA binding transcription factor activity, transferase activity, and nucleic acid binding. The most enrichment CC terms were intracellular, cell part, intracellular part, intracellular organelle, and membrane‐bounded organelle. Then Kyoto Encyclopedia of Genes and Genomes (KEGG) pathway analysis of the 270 mRNAs was performed to explore the signaling pathways involved. A total of nine significant pathways were enriched including insulin signaling pathway, pyrimidine metabolism, carbohydrate digestion and absorption, and pyruvate metabolism.

### 3.5 The screening of core lncRNAs during the proliferative phase of rat LR

**Figure 3:**
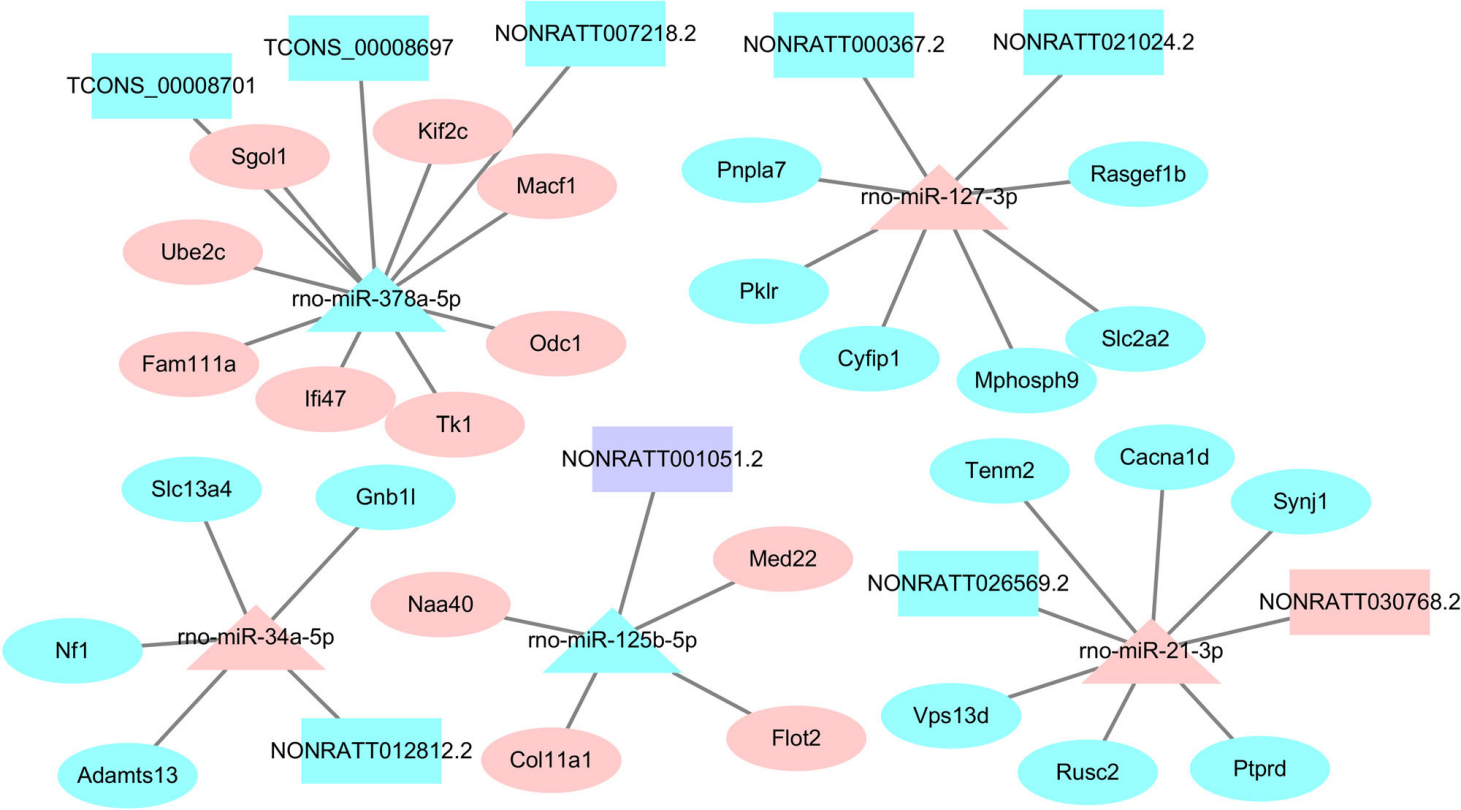
Core ceRNA network during the proliferative phase of rat LR. Rectangles, triangle, and ellipses represented DE lncRNAs, DE miRNAs, and DE mRNAs, respectively. Pink, light blue, and purple color represented upregulation, downregulation, and up/downregulation. CeRNA: competition endogenous RNA; DE: differently expressed; lncRNAs: long noncoding RNAs; miRNAs: microRNAs; mRNAs: messenger RNAs [Color figure can be viewed at wileyonlinelibrary.com]

The lncRNAs–miRNA–mRNA network was consisted with 107 lncRNAs, 32 miRNAs, and 270 mRNAs. Of these miRNAs, five were reported to play an important role during LR, including miR‐21, miR‐127, miR‐34a, miR‐378, and miR‐125b, and they were regarded as the core miRNAs. The core lncRNAs were selected with a differently expression, and they were associated with the five miRNAs. Finally, nine core lncRNAs were correspondingly identified (Figure 3).

### 3.6 TF–miRNA–lncRNA regulatory network

**Figure 4:**
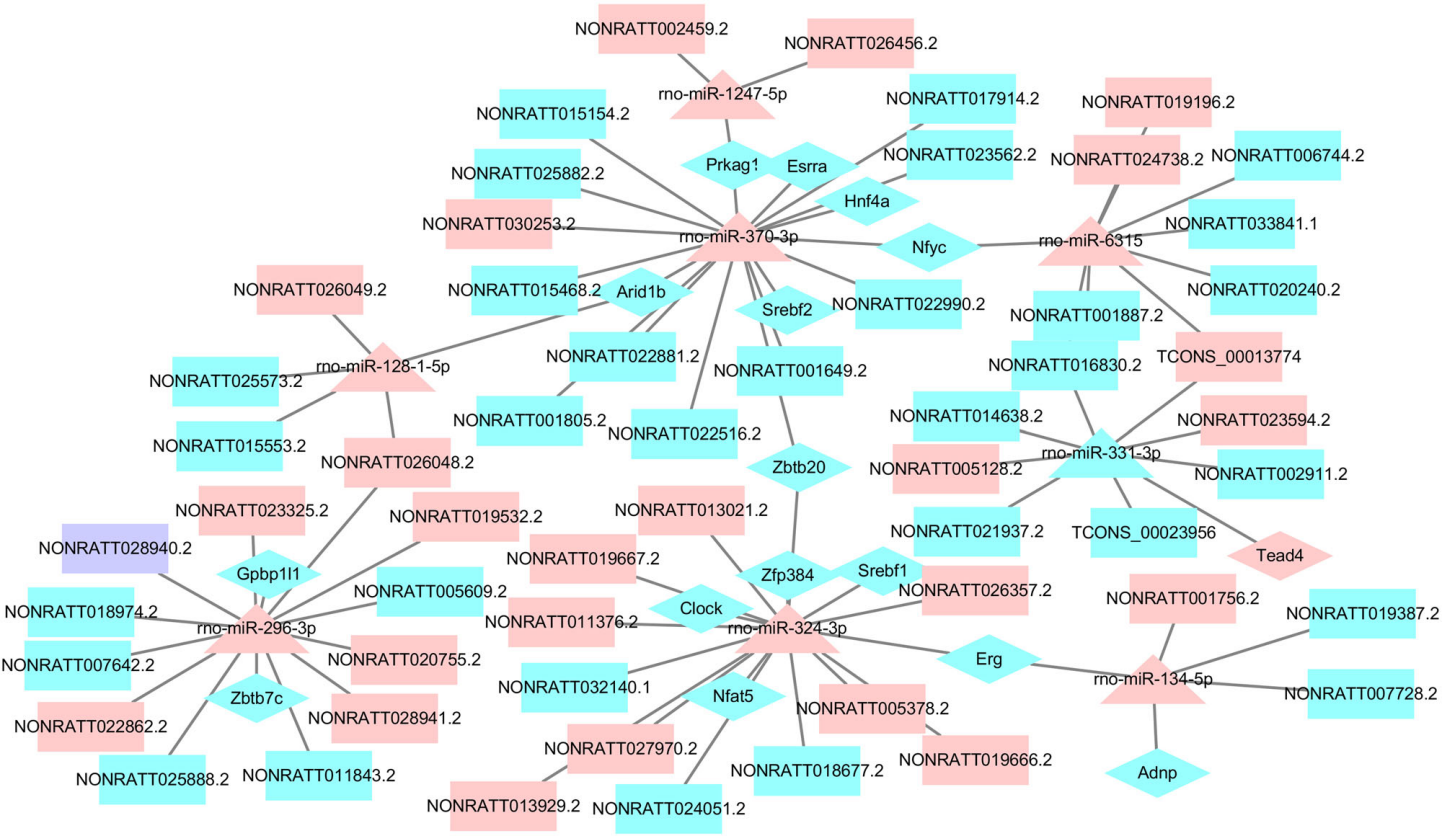
The regulatory network for TFs. Rectangles, triangle, and diamond represented DE lncRNAs, DE miRNAs, and TFs, respectively. Pink, light blue, and purple color represented upregulation, downregulation, and up/downregulation. DE: differently expressed; lncRNAs: long noncoding RNAs; miRNAs: microRNAs; TFs: transcription factors [Color figure can be viewed at wileyonlinelibrary.com]

TFs in the lncRNA–miRNA–mRNA network were analyzed using AnimalTFDB database. The result showed that 21 TFs (CSRNP1, MYBL2, TEAD4, EGR2, ESRRA, ARID1B, ZBTB20, NFYC, SREBF2, HNF4A, PRKAG1, NFAT5, SREBF1, ERG, ZFP384, CLOCK, RORC, MAFB, GPBP1L1, ZBTB7C, and ADNP) were identified to be involved, and then a TF–miRNA–lncRNA regulatory network was constructed (Figure 4).

## 4 DISCUSSION

Previous studies have shown that LR was regulated by a number of biological molecules including hormones, growth factors, and cytokines. However, most of these studies are limited to the protein‐coding genes, and it is still largely unknown how these genes are regulated during LR. Therefore, it is necessary to find new regulators involved in LR for better understanding the mechanism. Recent studies have shown that lncRNAs were important regulators of gene expression and associated with many important cellular physiological activities such as cell proliferation and differentiation (Ma et al., 2015; Zhu & Xu, 2013). LncRNAs could act as miRNA sponges to regulate the target mRNAs. The role of lncRNAs has been studied in a variety of cancer‐related diseases including HCC. Staff et al. identified two miRNAs (miR‐192 and miR204) could directly suppress lncRNA HOTTIP expression and interrupt GLS1‐mediated glutaminolysis in HCC (Staff, 2016). Chen et al. indicated that lncRNA PTENP1 could modulate cell proliferation, migration, autophagy, and apoptosis by decoying miR‐17, miR‐19b, and miR‐20a in HCC cell (C. L. Chen et al., 2015). However, the role of lncRNA–miRNA–mRNA network remains largely unknown during the proliferative phase of rat LR.

In this study, high‐throughput sequencing was conducted to analyze the expression changes of lncRNAs, miRNAs, and mRNAs during the proliferative phase of rat LR. Based on the RNA sequence data, 286 DE miRNAs, 1,738 DE lncRNAs, and 2,727 DE mRNAs were identified during the proliferative phase of LR compared with the normal group. Some DE miRNAs have been reported to be associated with LR. However, the function of most lncRNAs has not been studied. Then, an lncRNA–miRNA–mRNA interaction network was constructed during the proliferative phase of rat LR involving 107 lncRNAs, 32 miRNAs, and 270 mRNAs. To study the underlying role of lncRNAs during the proliferative phase of rat LR, GO enrichment analysis of the target mRNAs was performed. The result indicated that a large amount of significant GO terms were related to cellular metabolic process, cell adhesion, cellular response to stimulus, cell communication, and cell cycle, which had been reported as important physiological activities during the proliferative phase of rat LR (Erickson, Thompson, & Hixson, 2006; Kotsis et al., 2018; Loyer et al., 1994; Qin, Zhao, Chen, & Xu, 2006; Zheng, Weng, & Yu, 2009). KEGG pathway analysis identified nine signaling pathways including insulin signaling pathway and substance metabolism including pyrimidine metabolism, carbohydrate digestion and absorption, and pyruvate metabolism. Sasaki et al. indicated that insulin transmitted signal to intracellular regulators involved in hepatocyte growth through insulin receptor substrate 1 (IRS‐1) during rat LR (Sasaki, Zhang, Nishiyama, Avruch, & Wands, 1993). The substance metabolism could supply energy and materials for the synthesis of DNA and proteins during the proliferative phase of rat LR (Yin, Chang, & Xu, 2017). In the lncRNA–miRNA–mRNA interaction network, five core miRNAs (miR‐21‐3p, miR‐34a‐5p, miR‐127‐3p, miR‐378a‐5p, and miR‐125b‐5p) and nine core lncRNAs (NONRATT026569.2, NONRATT030768.2, NONRATT012812.2, NONRATT000367.2, NONRATT021024.2, NONRATT007218.2, TCONS_00008697, TCONS_00008701, and NONRATT001051.2) were identified according to the literature and regulation relationship between lncRNAs and miRNAs.

Some studies indicated that miR‐21 was upregulated and played a significant role in modulating cell cycle progression and hepatocyte proliferation by targeting PTEN, FASLG, CCND1, BTG2, and PELI1 during LR (Castro et al., 2010; X. Chen et al., 2016; Li, Chan, Leung, Wang, & Xu, 2015; Marquez, Wendlandt, Galle, Keck, & McCaffrey, 2010; Ng, Song, Roll, Frandsen, & Willenbring, 2012; Song et al., 2010). Thus, NONRATT026569.2 and NONRATT030768.2 might regulate cell cycle progression and hepatocyte proliferation to contribute to rat LR by interacting with miR‐21‐3p. A few studies demonstrated that miR‐34a was upregulated and was associated with the suppression of hepatocyte proliferation and cell apoptosis by targeting Notch receptors, BCL‐2, BCL‐XL, INHBB, and MET during LR (H. Chen et al., 2011; X. P. Wang et al., 2017). Therefore, NONRATT012812.2 might regulate hepatocyte proliferation and cell apoptosis to control rat LR by targeting miR‐34a‐5p. Pan et al. suggested that miR‐127 was downregulated and might facilitate hepatocyte proliferation by releasing BCL6 and SETD8 during rat LR (Pan et al., 2012). Hence, NONRATT000367.2 and NONRATT021024.2 might accelerate hepatocyte proliferation by regulating miR‐127‐3p. Song et al. discovered that miR‐378 directly inhibits ornithine decarboxylase (Odc1), which is known to promote DNA synthesis in hepatocytes after 2/3 PH (Song et al., 2010). So NONRATT007218.2, TCONS_00008697 and TCONS_00008701 might control hepatocyte proliferation during rat LR by interacting with miR‐378a‐5p. In this study, miR‐127‐3p was also predicted to target ENSRNOT00000079185 (ODC1), which was consisted with previous study. Hyun et al. showed that miR‐125b could contribute to liver regeneration by mediating Hedgehog signaling (Hyun et al., 2015). It suggested that NONRATT001051.2 might be conducive to liver regeneration by targeting miR‐125b‐5p.

Many TFs have been reported during rat LR including E2F2, KLF2, STAT3, NFkappaB, AP‐1, C/EBPbeta, and Nrf2. In this study, 21 transcription factors (CSRNP1, MYBL2, TEAD4, EGR2, ESRRA, ARID1B, ZBTB20, NFYC, SREBF2, HNF4A, PRKAG1, NFAT5, SREBF1, ERG, ZFP384, CLOCK, RORC, MAFB, GPBP1L1, ZBTB7C, and ADNP) were found to be involved in TF‐miRNAs regulation network during the proliferative phase of rat LR. Zinc‐finger protein ZBTB20, also named DPZF, HOF, and ZNF288, was a critical regulator of EGFR expression and hepatocyte proliferation in mouse liver regeneration (H. Zhang et al., 2018). HNF4α, a member of the nuclear receptor family of transcription factors, could maintain hepatocyte differentiation in the adult healthy liver, and its loss may directly contribute to hepatocellular carcinoma development (Bonzo, Ferry, Matsubara, Kim, & Gonzalez, 2012). CLOCK, belonging to the bHLH‐PAS family, located in the cell nucleus, played an important role in the regulation of liver gene expression (Malatesta, Baldelli, Marcheggiani, & Gazzanelli, 2003). The nuclear factor of activated T‐cells (NFAT) transcription factors represented a family of gene transcription signaling intermediates that translate receptor‐dependent signaling events into specific transcriptional responses using the Ras/Raf pathway, and NFAT4 played an important role in liver regeneration (Pierre et al., 2009). However, the function of most TFs was still unclear.

Some limitations were existed in this study. LncRNAs have a variety of functions. However, only the role of lncRNAs as miRNA sponges was analyzed through building the regulatory network of lncRNA–miRNA–mRNA and lncRNA–miRNA–TF. In addition, key lncRNAs predicted by bioinformatics analysis were not experimentally verified during the proliferative phase of rat LR.

## 5 CONCLUSIONS

First, DE lncRNA, DE miRNA, and DE mRNA were analyzed by high‐throughput sequencing technology, and then the lncRNA–miRNA–mRNA regulatory network was constructed according to the regulation mechanism of lncRNAs. Finally, through literature review and lncRNA–miRNA regulatory pairs, nine key lncRNAs, and five key miRNAs were screened out, which may play an important role during the proliferative phase of rat LR. This study provided clues for revealing the mechanism of LR and offered new ideas for the treatment of liver‐associated diseases

## PUBLICATION ETHICS

All experimental procedures in this study were approved by and performed according to the guidelines for the care and use of experimental animals that have been established by the Animal Care and Use Committee of the Ministry of Science of the People's Republic of China.

## CONFLICT OF INTERESTS

The authors declare that there are no conflict of interests.

## AUTHORS CONTRIBUTION

Haijing Bai wrote the manuscript and analyzed data. Cunshuan Xu participated in its design and interpretation. Wei Jin, Jianlin Guo, Xueqiang Guo, and Cuifang Chang critically revised the manuscript, and all authors have read and given approval of the manuscript.

## DATA SHARING AND DATA ACCESSIBILITY

The data that support the findings of this study are available from the corresponding author upon reasonable request.

## Supporting information

Supporting information

Supporting information

Supporting information

Supporting information

Supporting information

Supporting information

Supporting information
