# Supplementary figures and images for: Comprehensive analysis of lncRNA–miRNA–mRNA during proliferative phase of rat liver regeneration
Source: J Cell Physiol. 2019 Mar 27;234(10):18897–905. doi: 10.1002/jcp.28529 (PMC6617821; doi:10.1002/jcp.28529)

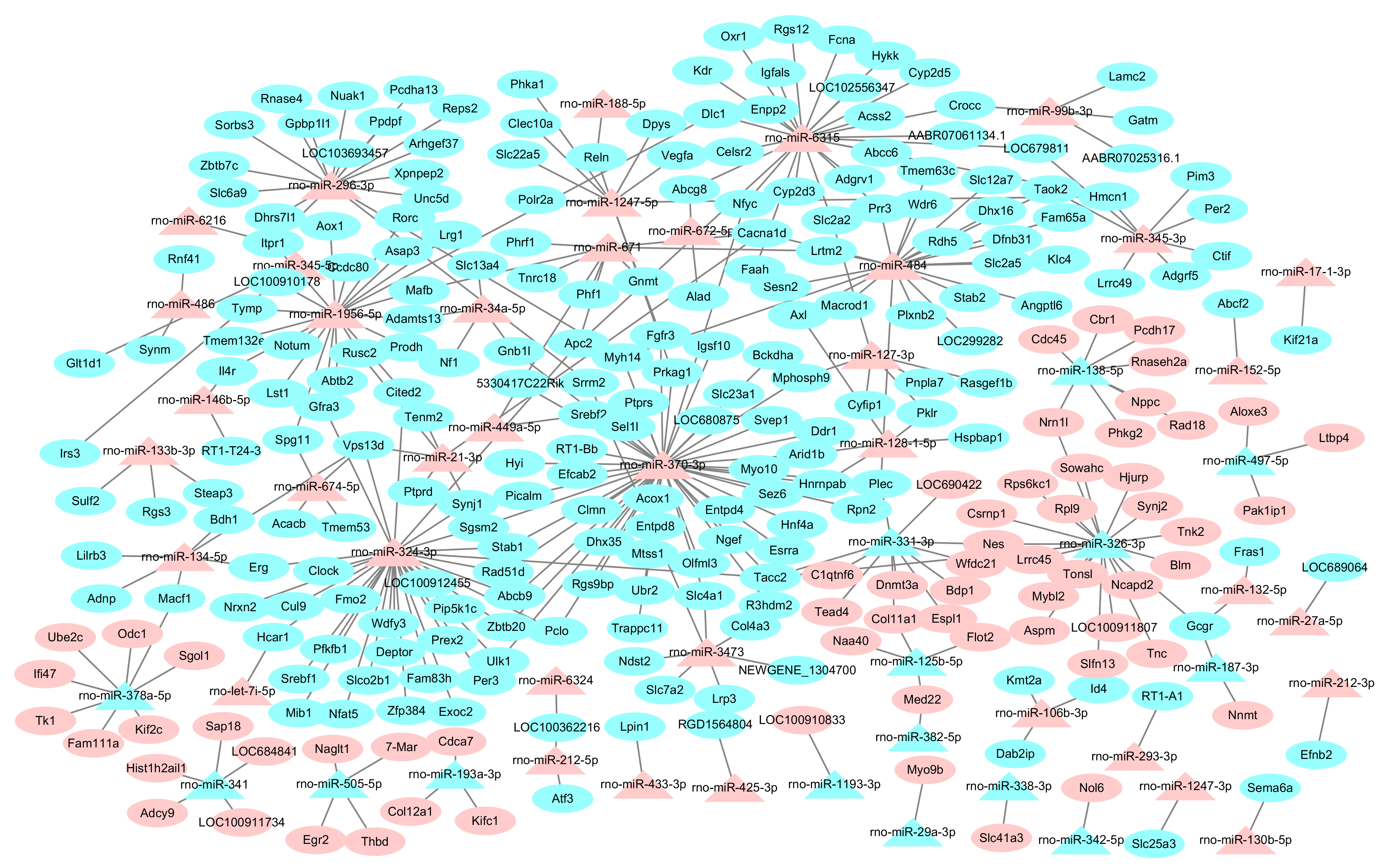

Supplement: Supplementary file 4 — Supporting information [file JCP-234-18897-s004.tif]

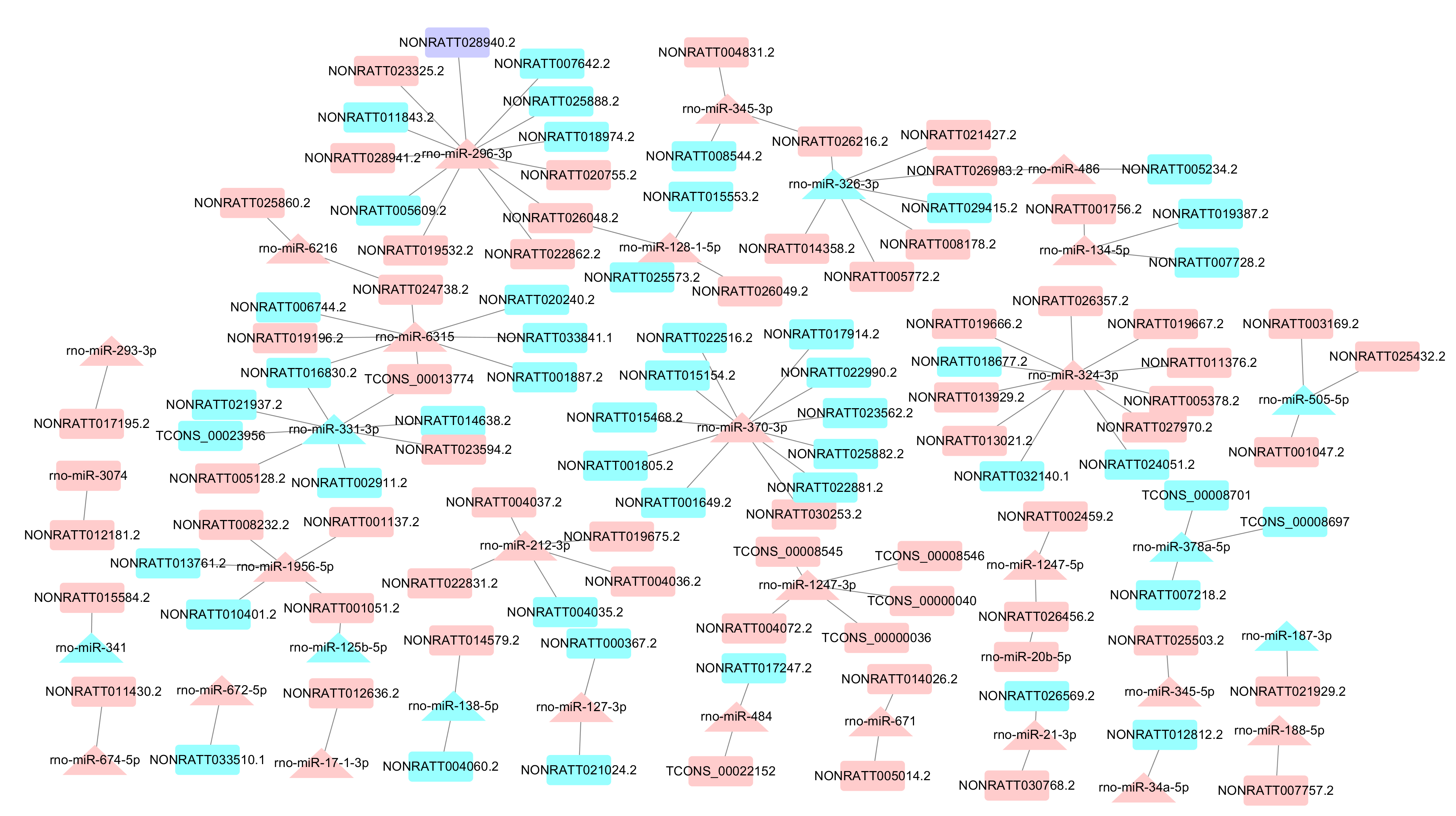

Supplement: Supplementary file 5 — Supporting information [file JCP-234-18897-s005.tif]
