# Supplementary material for: Comprehensive analysis of lncRNA–miRNA–mRNA during proliferative phase of rat liver regeneration
Source: J Cell Physiol. 2019 Mar 27;234(10):18897–905. doi: 10.1002/jcp.28529 (PMC6617821; doi:10.1002/jcp.28529)
Supplement: Supplementary file 7 — Supporting information [file JCP-234-18897-s007.docx]

# **Appendices**

# Additional file 1: The results of analyzing differentially expressed lncRNAs at 12h, 24h, 30h, 36h, and 72h after PH compared with CG.

# Additional file 2: The results of analyzing differentially expressed miRNAs at 12h, 24h, 30h, 36h, and 72h after PH compared with CG.

# Additional file 3: The results of analyzing differentially expressed mRNAs at 12h, 24h, 30h, 36h, and 72h after PH compared with CG.

# Additional file 4: DEmiRNA-DEmRNA interaction networks during the proliferative phase of rat LR. Triangles and ellipses represented DE miRNAs and DEmRNAs. Pink and light blue color represented up-regulation and down-regulation

# Additional file 5: DEmiRNAs-DElncRNAs interaction networks during the proliferative phase of rat LR. Triangles and rectangles represented DE miRNAs and DElncRNAs. Pink, light blue and purple color represented up-regulation, down-regulation and up/down regulation.

# Additional file 6: GO enrichment analysis of mRNAs in the ceRNA network.
